# Supplementary material for: A cascading model for nudging employees towards energy-efficient behaviour in tertiary buildings
Source: PLoS One. 2024 May 16;19(5):e0303214. doi: 10.1371/journal.pone.0303214 (PMC11098420; doi:10.1371/journal.pone.0303214)
Supplement: S1 File — Validation Profile Questionnaire. (PDF) [file pone.0303214.s001.pdf]

Part 1.a: Profile: General information - socio-demographics

- **1.a.1 Age group:**
  - ☐ <21
  - ☐ 22-40
  - ☐ 41-52
  - ☐ 53-71
  - ☐ 72+

- **1.a.2 Gender:**
  - ☐ Female
  - ☐ Male
  - ☐ Other

- **1.a.3 Education:**
  - ☐ None
  - ☐ High-school /secondary
  - ☐ Post-secondary (non-university)
  - ☐ University degree (bachelor or equivalent)
  - ☐ Post-graduate (master or equivalent)
  - ☐ Doctoral degree (PhD or equivalent)

- **1.a.4 Country:**
  - ☐ Spain
  - ☐ Austria
  - ☐ Greece
  - ☐ UK
  - ☐ Other

Part 1.b: Profile: Employment profile

- **1.b.1 Which of the following best describes your office/ team/ department culture:** (you can check only one option)
  - ☐ Teamwork, participation, sharing
  - ☐ Get the job done and goal-oriented
  - ☐ Encourage creativity, experimentation and risk taking
  - ☐ Formal and hierarchical (I work on my own)
  - ☐ None of them

Part 1.c: Energy efficiency at work

- **1.c1 Assume that the organisation you work for has just launched an initiative to reduce energy consumption. You can join on a voluntary basis and save energy by changing your behaviour in line with**

the simple tips and instructions you receive. What would you do?

- ☐ Join immediately
- ☐ Discuss first with colleagues and then decide
- ☐ Join after a while
- ☐ Do not participate

- **1.c.2 I wish to further contribute to energy efficiency in the building; However, in practice (Please, select the statement which is most applicable to you):**
  - ☐ I often neglect or do not remember it, as I am preoccupied with other work activities.
  - ☐ I am not sure about what is a good energy practice so I do little or nothing.
  - ☐ I am discouraged by the attitude of my colleagues and/or of the management, so I do little or nothing.
  - ☐ None of the answers above apply to me.

Part 1.d: User behaviour Type (Pinball-Shortcut-Thoughtful)

- **1.d.1 Please, evaluate the next statements related to energy management at work**

	Strongly disagree	Disagree	Neither agree or disagree	Agree	Strongly agree
I make 'good enough' choices rather than expending effort in trying to 'optimise' my energy-related choices.	☐	☐	☐	☐	☐
I do the same thing each time and the same stimulus is applied to me in relation to energy consumption. I do not think too much about any decisions related to energy. I focus on doing my work and no more.	☐	☐	☐	☐	☐
In relation to energy consumption I think about what I do and I try to provide the 'why' analytically. I set and modify my own goals. I learn from mistakes (mine and those of others) and I change my behaviour accordingly.	☐	☐	☐	☐	☐
I hardly pay attention to physical changes or notifications at my workplace that are not directly related to my work	☐	☐	☐	☐	☐
I prefer following my peers to take decisions	☐	☐	☐	☐	☐
I look for data to take my everyday decisions	☐	☐	☐	☐	☐

I prefer full system automation than taking decisions	☐	☐	☐	☐	☐
Prompts are what work for me to take action	☐	☐	☐	☐	☐
I don’t see myself following an advice from others without self-reflecting in advance	☐	☐	☐	☐	☐

Part 2: Self-assessment of energy-related Pro-environmental Intentions (Precontemplation-Contemplation-Action) and Confidence in Technology

- **2.1 Please, evaluate these statements:**
  - I am actually changing my energy intensive habits and saving energy right now.
    ☐ Strongly disagree ☐ Disagree ☐ Neutral ☐ Agree ☐ Strongly Agree
  - My behavioural choices sometimes have a negative impact on energy efficiency.
    ☐ Strongly disagree ☐ Disagree ☐ Neutral ☐ Agree ☐ Strongly Agree
  - Modern science will solve our energy-related problems.
    ☐ Strongly disagree ☐ Disagree ☐ Neutral ☐ Agree ☐ Strongly Agree
  - It is a waste of time thinking about energy savings.
    ☐ Strongly disagree ☐ Disagree ☐ Neutral ☐ Agree ☐ Strongly Agree
  - I enjoy living as I please, but sometimes my behaviours are harmful to the energy efficiency.
    ☐ Strongly disagree ☐ Disagree ☐ Neutral ☐ Agree ☐ Strongly Agree
  - Sometimes I think I should cut down on my wasteful behaviour.
    ☐ Strongly disagree ☐ Disagree ☐ Neutral ☐ Agree ☐ Strongly Agree
  - I am at the stage where I should think about being more active in reducing energy consumption.
    ☐ Strongly disagree ☐ Disagree ☐ Neutral ☐ Agree ☐ Strongly Agree
  - I have just recently changed my environmentally energy related harmful habits.
    ☐ Strongly disagree ☐ Disagree ☐ Neutral ☐ Agree ☐ Strongly Agree
  - I don't think I behave in ways that cause too much harm to the energy efficiency.
    ☐ Strongly disagree ☐ Disagree ☐ Neutral ☐ Agree ☐ Strongly Agree
  - Trying to live in a more energy sustainable manner would be pointless for me.
    ☐ Strongly disagree ☐ Disagree ☐ Neutral ☐ Agree ☐ Strongly Agree
  - I am trying to engage in less environmentally energy-related harmful behaviours than I used to.
    ☐ Strongly disagree ☐ Disagree ☐ Neutral ☐ Agree ☐ Strongly Agree
  - With respect to the energy efficiency, there is no need for me to think about changing my daily behaviours.
    ☐ Strongly disagree ☐ Disagree ☐ Neutral ☐ Agree ☐ Strongly Agree
  - Anyone can talk about wanting to do something about the energy efficiency, but I am actually doing something about it.
    ☐ Strongly disagree ☐ Disagree ☐ Neutral ☐ Agree ☐ Strongly Agree

Part 3: Ranking of best Persuasion Strategies

- **3.1 Top 10: Please order the following strategies from 1 to 10 as incentives that could engage you to save energy based on your experience through the GreenSoul project. 1 refers to the most preferable and 10 to the least preferable option for you..**

**Strategy**	**Principle**	**Order of preference given by respondent**
Public (social) recognition of your contribution to energy savings is provided.	Social Recognition	
Receive energy related information in a simple and aesthetically appealing way.	Physical attractiveness	
Receiving perks such as flexible working hours, skipping certain tasks, etc, as a reward for improving your energy performance.	Conditioning	
You and your team find out ways to collectively achieving energy savings.	Reciprocity	
Your (top) managers are also committed to save energy.	Authority	
You can monitor & track your own energy performance in real-time. Historical comparison of your energy performance and/or consumption is provided.	Self-monitoring	
Information on the actual effect that your (potential) actions may have upon the energy consumption	Cause & Effect	
Tips or suggestions on the energy saving practice of the day/ week	Suggestion	
Progress, tips and lessons learned on specific energy saving actions performed by other users that are similar to me	Similarity	
Knowing that most of your fellow community colleagues do try to conserve energy at workplace.	Social Proof	
