# Supplementary material for: A cascading model for nudging employees towards energy-efficient behaviour in tertiary buildings
Source: PLoS One. 2024 May 16;19(5):e0303214. doi: 10.1371/journal.pone.0303214 (PMC11098420; doi:10.1371/journal.pone.0303214)
Supplement: Attachment — Submitted filename: Rebuttal paper PLOS-ONE.pdf [file pone.0303214.s002.pdf]

Responses to reviewers

First of all, we would like to express our gratitude to the editors and the external reviewers for their valuable and constructive comments, which we found most helpful in improving the overall quality of our paper toward the final version.

We acknowledge the opportunity to submit a new version of this manuscript and now we think it can be considered as final. Thus, we have carefully examined the comments from all reviewers and followed their suggestions. We believe we have addressed all their points in light of their suggestions and comments, which are summarised in the authors' responses below.

To facilitate the reviewing task, we have made all the changes to the manuscript in different colours to differentiate the edited part; **blue** for new additions and **purple** for edited or transformed parts. We refer to them as well in our responses.

Reviewer 1

Overall, the paper needs significant improvements on the following aspects:

1. The literature review of this article is very terse. Many recent studies discussing energy consumption behavioral change has not been discussed in the literature review. The authors should elaborate more on this aspect by discussing the following studies: Smart Sensing and End-User Behavioral Change in Residential Buildings: An Edge Internet of Energy Perspective; Is there any room for renewable energy innovation in developing and transition economies? Data envelopment analysis of energy behaviour and resilience data; The emergence of explainability of intelligent systems: Delivering explainable and personalized recommendations for energy efficiency; Intelligent edge-based recommender system for internet of energy applications; Understanding Waste Management Behavior Among University Students in China: Environmental Knowledge, Personal Norms, and the Theory of Planned Behavior; A novel approach for detecting anomalous energy consumption based on micro-moments and deep neural networks; Reshaping consumption habits by exploiting energy-related micro-moment recommendations: A case study; REHAB-C: Recommendations for Energy HABits Change; A micro-moment system for domestic energy efficiency analysis; Using big data and federated learning for generating energy efficiency recommendations; Smart fusion of sensor data and human feedback for personalized energy-saving recommendations; IoT based smart and intelligent smart city energy optimization.

Thank you very much for your valuable suggestion. We acknowledge the necessity to expand our literature review to incorporate recent studies addressing energy consumption behaviour

change. In response to your suggestions, we have revised the **Introduction** section to include key studies that align with our research scope.

Among others, we have integrated references like "Smart Sensing and End-User Behavioral Change in Residential Buildings: An Edge Internet of Energy Perspective" and "Is there any room for renewable energy innovation in developing and transition economies?" to emphasize the evolving landscape of energy consumption and efficiency. Additionally, we have discussed studies focusing on "The emergence of explainable intelligent systems" and "Intelligent edge-based recommender systems for the Internet of energy applications" to underline the advancements in personalized and intelligent energy-saving systems.

Moreover, we have included insights from "Understanding Waste Management Behavior Among University Students in China" and "A novel approach for detecting anomalous energy consumption" to showcase diverse aspects influencing energy behaviour. The inclusion of studies on micro-moments and federated learning further enriches our discussion on innovative approaches to energy efficiency.

These additions provide a more comprehensive view of the current state of research in energy-saving behaviours and recommendation systems. We believe that these enhancements significantly improve the literature that contextualizes our manuscript.

2. Kindly fix the formatting problem in page 2..
Thank you for bringing this to our attention. The manuscript has been thoroughly reviewed to address potential issues.

3. While the sample size of 743 users across four countries is commendable, the authors should discuss the extent to which the findings are generalizable to wider populations, especially outside Europe.
We can not agree more with the reviewer's assessment. The concern regarding the self-reported nature of data introducing biases is valid and acknowledged. For this reason, in the "Limitations of the current work" section, the manuscript addresses various constraints, including sample size and population diversity, which may affect the generalizability of the results across different societies and cultures. The limitation concerning the context-specific factors/features for training the models and their dependency on energy efficiency in the workplace is also discussed. Additionally, the manuscript recognizes the subjective nature of user-reported appropriateness of persuasion principles and the challenges in evaluating their actual effectiveness. Future research directions are proposed to address these limitations, including expanding the study to different contexts and linking the persuasion strategies to well-established behavioral frameworks.

Nevertheless, the **"Limitations of the current work"** section of the manuscript has been updated to acknowledge this concern more explicitly:

> The diversity in energy behaviors and cultural differences beyond these four European countries limits the extent to which our findings can be applied globally. Therefore, caution must be exercised in generalizing our results to populations in diverse geographic and socio-cultural contexts outside of Europe. This limitation underscores the need for further research in varied settings to enhance the universality of our findings. More particularly, provides us with a line for future research to remove the location factor (country) and explore if our computational model works better when people belong to the same society/culture.

4. The self-reported nature of the data can introduce biases. The authors might consider addressing this aspect and discussing the potential limitations of the self-reported data.

Once again, we fully agree with the reviewer's suggestion. Self-reporting allows participants to provide information based on their personal experiences and perceptions, which is vital in behavioural studies like ours that focus on subjective aspects such as personal attitudes and beliefs. While self-reported data can be influenced by biases, it offers valuable insights into individual behaviours and motivations that are often difficult to measure through observational methods alone.
In any case, we recognize the limitations of using self-reported data in our study and, in response to their recommendation, we have revised the "Limitations of the current work" section to more explicitly address this concern:

> Additionally, the subjective nature of the self-reported data in our study is a significant limitation, as it can introduce biases related to personal perceptions, potentially affecting the accuracy and reliability of our findings. This aspect is crucial in understanding the implications and applicability of our results.

5. The paper briefly mentions increased accuracy over non-personalized methods but lacks an in-depth comparison or analysis. This comparison would be beneficial for readers to gauge the actual improvement the proposed framework offers.
Once again, thanks for spotting this important aspect in the comparison of the proposed methods. This is true that we increased the accuracy of the non-personalised methods in our fusion approach. However, we might have overlooked a better explanation of what we call non-personalised methods. A non-personalised method can provide a new user with a random persuasive strategy and see how this user reacts to this cue. Another option, potentially more tailor-made, but still not bespoke to a specific user, is to provide a newcomer with the persuasive strategy rated as the top for the majority of the respondents of our dataset. In this particular case, we compared our method against such a non-personalised baseline with the F-score in Tables 4 and 5. Recall that the top-rated persuasive strategies were: 1. “Conditioning”, 2. "Social-recognition", 3. "Physical attractiveness", 4. “Self-monitoring”, and 5. “Reciprocity”. Therefore, in the mentioned tables the F-score is calculated with the Conditioning principle in most of the cases. We hope that this explanation will help the reviewer and potential

readers to better understand the comparison method. A similar explanation is provided in the manuscript (Section: “Fusion of optimal ranking and score prediction”). Also, a new Figure has been added to explain the top principles.

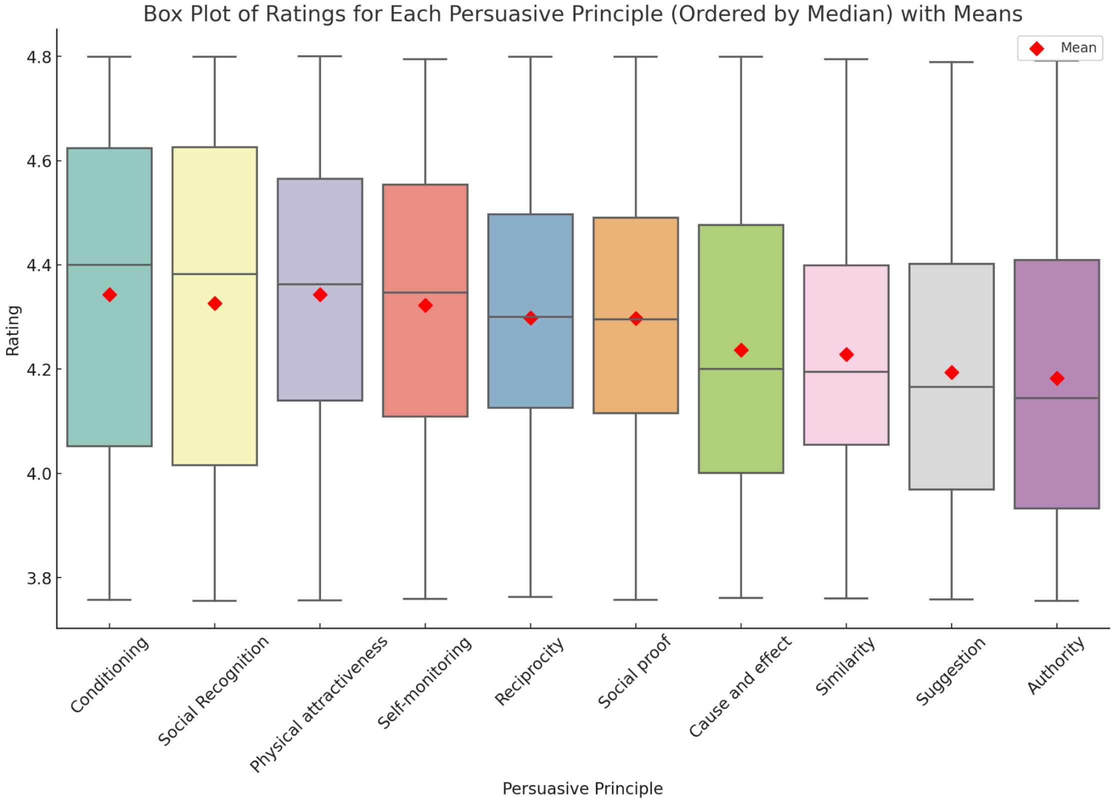

6. The conclusion highlights the potential of the framework, but there is a lack of discussion about the practical challenges or considerations in deploying such a system in real-world settings.

Thank you for your insightful feedback and for highlighting the importance of discussing the practical challenges and considerations in deploying our proposed framework in real-world settings. We appreciate your attention to this critical aspect of our research.

We agree that the practical application of any theoretical framework is a crucial component of its overall evaluation and impact. To address this, we have indeed included a discussion on the practical challenges and considerations of deploying our system in the "Applying the model in real-world scenarios" subsection of our Discussion section. In this subsection, we have attempted to provide a comprehensive overview of the potential real-world applications and the associated challenges, including aspects such as data collection, user privacy, ethical considerations, and the adaptability of our approach to different contexts and user profiles.

However, we acknowledge that these points may not have been as prominent or as detailed as necessary. We are grateful for your comment as it provides us with an opportunity to revisit this section and ensure that the discussion on practical deployment challenges is adequately highlighted and elaborated upon.

In essence, it is important to address the practical challenges and considerations that may arise during the implementation of our proposed framework in real-world settings. Firstly, integrating our system into existing infrastructures requires careful planning and adaptation to ensure compatibility with different technological environments across the workspace. Secondly, collecting and handling user data, which is a cornerstone of our framework, must navigate the complex landscape of privacy laws and ethical considerations, as we will explore in the next sub-section. Thirdly, the effectiveness of our system depends on user engagement and acceptance. Additionally, the scalability of the system must be considered to ensure that it remains efficient and effective as it adapts to varying numbers of users and different environments. Finally, ongoing monitoring and evaluation are necessary to identify and address any unforeseen issues after deployment, and to ensure the system's relevance and effectiveness in dynamically changing real-world scenarios.

Reviewer 2

1. I think the contribution to the existing body of literature of this work seems too limited. The work extends the authors' own previous works, and the main addition seems to lie in the combination of two prediction models (and some additional data that is collected). The outcome of the combination is positive. For a journal publication, I would expect a much deeper analysis. I appreciate that the authors discuss limitations of their work in various respects. Still, I would have hoped that the paper delivers more than trying out some combination of predictors on a dataset (which also has some major limitations).

Thank you for your insightful comments and for recognising the positive outcomes of our research. We appreciate your perspective regarding the scope and depth of our analysis. Our work, indeed, builds upon our previous research, integrating two prediction models and incorporating additional data to enhance the understanding of energy efficiency in buildings. This approach, while seemingly straightforward, addresses a critical gap in the existing literature by offering a novel, practical solution that can be readily implemented in real-world scenarios. The combination of these models, as demonstrated in our study, provides a more robust and accurate framework for energy efficiency prediction than what was previously available. This integration is not just a mere aggregation of predictors but a methodical fusion that leverages the strengths of each model to overcome the limitations inherent in using them separately.

We acknowledge the limitations of our dataset, as highlighted in the paper. However, these constraints also reflect the realistic challenges encountered in this field of research. Addressing these limitations within the scope of this study requires starting the research from scratch, which is beyond the feasibility of our current project timeline and resources.

Moreover, acknowledging these limitations in our paper serves as a foundation for future research, prompting further exploration and refinement in this area. We believe our work contributes significantly to the body of literature by not only presenting a novel approach but also by laying the groundwork for future advancements.

2. Unfortunately, the authors decided to not share the code and the data, which makes it impossible for others to validate the findings. The authors argue that they do not want to do this for privacy/ethical reasons, but it remains unclear why it is impossible to a) share the code for the analyses and b) share anonymized data.

Thank you for your insightful comment. For the sake of replicability and open research, we have released the datasets.

Two already exist from GreenSoul project (already cited in the manuscript):

- https://zenodo.org/records/2610102
- https://zenodo.org/records/3565757

A new dataset with all the evaluated features and with data from prolific and Greensoul project together. The data is already codified in JSON for the sake of using this dataset on other projects in an easy way.

- https://doi.org/10.5281/zenodo.10377229

The GitHub with all the methods for the recommender system is here:

- https://github.com/morelab/st_recommender/tree/main

3. There is some difference between nudging and persuasion, according to the literature. Should be discussed..

Thank you once again for your feedback. This is indeed something important for the readers of the article and we used both terms in our manuscript. To cope with this important aspect, we have created a new subsection in the Discussion section. This is the content:

> While persuasion in literature employs strategic communication to influence beliefs and actions, nudging subtly steers behaviour in a desired direction without necessarily applying direct persuasion. Both persuasion and digital nudging involve personalisation and present new opportunities for enhancing user experiences and bespoke interaction, though. Persuasion, in its broader sense, is an art of rhetoric, using specific formations to achieve desired effects on audiences. This can involve both rational and emotional appeals, influencing individuals' knowledge, beliefs, and interests. Nudging, on the other hand, is identified as a distinct mode of governance, aiming to modify behaviour subtly without explicit persuasion strategies or incentives such as pecuniary ones. In this manuscript, we are focused on persuasion principles and strategies to influence energy-behaviour activities. However, our approach indeed has a lot of similarities with nudging, as we are also trying to enact certain behaviours in a bespoke manner.

4. It would be helpful if the distribution of top-1 ranked strategies in the original data would be shown. In general, a deeper analysis of the underlying data seems advisable.

Thank you for identifying these valuable suggestions. Indeed we did not provide a full explanation. Therefore, a new Figure and explanation is created in the subsection Persuasion Principles (Materials and Methods) to list the top-ranked Persuasive principles. This is also linked with the dataset we released open in Zenodo. Please, see a glimpse of the top-ranked principles of persuasion from the whole sample in a boxplot way with means as well.

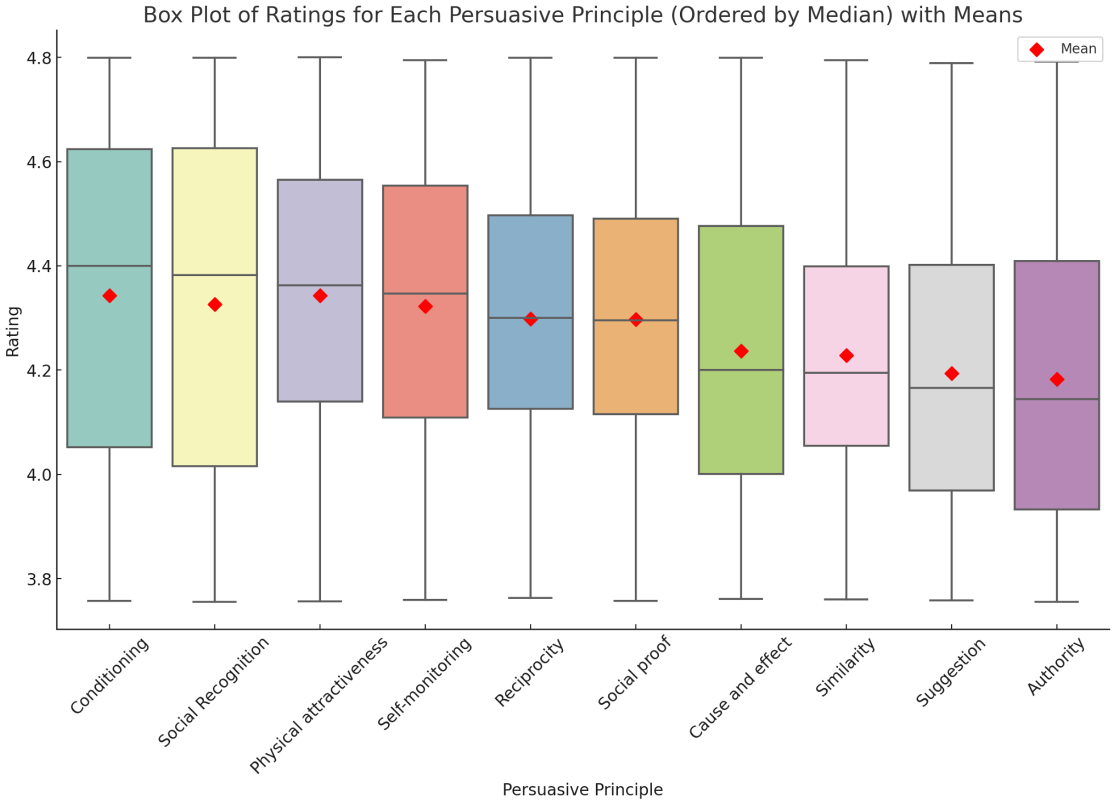

5. The data itself, as mentioned by the authors, might be rather noisy or even biased, depending, e.g., how things were interpreted by the participants.

Thank you for identifying these valuable minor changes suggested below. Self-reported data, while offering valuable subjective insights, can indeed be influenced by how participants interpret questions or by their own biases. Nonetheless, it offers valuable insights into individual behaviors and motivations that are often difficult to measure through observational methods alone.

For this reason, We have diligently included a comprehensive discussion in our manuscript about these limitations, emphasizing the need for cautious interpretation of our results. This approach aims to provide a balanced view of the study's findings, acknowledging both their value and their inherent constraints in the "Limitations of the current work" section:

Additionally, the subjective nature of the self-reported data in our study is a significant limitation, as it can introduce biases related to personal perceptions, potentially affecting the accuracy and reliability of our findings. This aspect is crucial in understanding the implications and applicability of our results.

6. Pre-processing and feature selection: The authors perform feature selection for a given classification task, but the classification task is not explicitly described.

Thank you for identifying this important aspect. We have diligently addressed this point. To reduce the features in the dataset, we obtained each user's top persuasive principle. Therefore, the top-1 persuasion principle was a categorical variable for each user.

That is, what we did was to understand which is the minimal number of user metrics that help us to predict which category a user falls into. In essence, this was not a classification activity per se, but a way to find intrinsically which were the user features that contribute the most (weights) to the objective of obtaining the top-1 principle (a categorical variable).

7. The threshold of 80% of keeping data seems a bit arbitrary.

Thank you for observing the decision to retain features that provide 80% of the information about users in our dataset. We understand your concern about this threshold appearing arbitrary and would like to offer a detailed rationale for our choice.

Our decision to use the 80% threshold for information retention in the dimensionality reduction process was guided by a balance between maintaining sufficient data integrity and ensuring computational efficiency. This approach is grounded in the Pareto Principle, often referred to as the 80/20 rule, which is a common heuristic in data science and many other fields. The principle suggests that approximately 80% of effects come from 20% of causes. In the context of our research, this translated to retaining the most significant features that contribute the majority of relevant information.

The following considerations drove the specific choice of 80%. In our study, we determined that preserving 80% of the total information successfully captured essential aspects of user behavior and preferences, without a significant loss in data integrity. This level of dimensionality reduction allowed us to concentrate on the most impactful features, thereby enhancing the relevance and accuracy of our analysis.

Maintaining all features in the dataset might have led to complications like overfitting and increased computational demands. By reducing the dimensionality to retain 80% of the information, we were able to make our model more computationally efficient, without greatly compromising the quality of our predictions. This approach is in line with previous research, where reducing dimensions to a principal subset of features, often around 80%, has proven effective in balancing information retention with model performance. Furthermore, we applied this threshold strategically in the design of our Prolific survey. By focusing on only the nine most critical user features, we kept the survey both concise and relevant. This approach helped in avoiding respondent fatigue and ensured higher response accuracy and participation rates.

While the threshold may seem arbitrary at first glance, it was a deliberate choice based on empirical evidence and best practices in data analysis. We believe this approach strikes an optimal balance for our study's objectives and contributes to the robustness and applicability of our findings.

8. "which are then fused sequentially to get aggregated results" -> it is not so clear at this stage what "fused sequentially" would mean..

We appreciate the reviewer's feedback and understand the concern regarding the phrase 'fused sequentially' in our manuscript. To clarify, in this context, 'fused sequentially' means that we combine the results from the two methods one after the other in a specific order. Essentially, we first obtain results from the first method and then incorporate the results from the second method in a step-by-step fashion to generate aggregated recommendations. To make this clearer for our readers, we propose revising the original text as follows:

> Based on the structure of these datasets, two different approaches, originally developed by the authors using preliminary data in previous publications, have been followed to identify the appropriateness of each persuasion strategy per user, each resulting in its recommendations, subsequently, these two sets of recommendations are merged in a step-by-step fashion, resulting in aggregated results. The first method, called here "Persuasion Principle Optimal Ranking", is based on an extended version of matrix factorization (MF) through Factor Machines based on the library LightFM . Specifically, it extends MF by including the latent factors of users (socio-economic features) and items (persuasive principles behind each strategy). This model results in personalised rankings of the selected persuasion strategies from the most appropriate for a user to the least appropriate. The second method, called here ``Persuasion Principle Score Prediction", is based on Random Forest regression and results in personalised predictions of the score that a user would give to a persuasion principle on a scale from one to five. The former model can predict the order in which a user prefers to receive a persuasive message (ranking), and the latter provides the impact that this persuasive message will have on the specific user through ratings.

9. "Specifically, it extends MF by including the latent factors of users (socio-economic features) and items" -> The paper is not self-contained here. It is also not clear at this stage what the elements of the matrix are which should be factorized..

Thank you for your feedback on our paper. Your comment has highlighted an area that requires further clarification. We agree that the section discussing the extension of matrix factorization (MF) with latent factors of users and items could be more explicit. To address this, we propose the following revision to make our paper more self-contained and clear:

Specifically, our approach extends traditional matrix factorization (MF) by incorporating latent factors that represent both users and items. For users, these latent factors are derived from socio-economic features, such as age, income level, and occupation, as specified in Table 2. For items, the latent factors correspond to the characteristics of the persuasive strategies being recommended, like their complexity, type, and targeted behaviour change. The elements of the matrix to be factorized are the interactions between users and items, quantified based on the users' responses to different persuasive strategies. This extended MF approach allows us to capture the nuances of user preferences and strategy effectiveness, leading to more personalized and effective recommendations.

By referencing Table 2, we provide a direct link to the specific socio-economic features used to generate the user latent factors, enhancing the clarity and comprehensiveness of our paper. This addition ensures that readers can easily find the detailed information on user features, further improving the paper's self-contained nature.

10. Fusion: A variety of list combination strategies are possible. Why was this particular fusion strategy applied?.

We thank the reviewer for raising this point, which we also believe is paramount to clarify. We chose the cascade fusion strategy for our model based on its ability to effectively combine different types of outputs that are not harmonized among them in a sequential manner: the optimal ranking and score prediction. The first method's ranking acts as a filter, narrowing down the top persuasion strategies, which are then fine-tuned using the second method's score predictions. This sequential fusion optimizes the recommendation process, ensuring a more accurate and tailored approach for each user. Besides, it's akin to a dimensionality reduction approach, simplifying the model's complexity while maintaining robust predictive power[1]. This strategy aligns with similar methodologies in previous research but with the added advantage of applying to categorical variables directly. This way, this strategy allows us to leverage the unique strengths of each model. The first model in the cascade acts as a filter, selecting the most relevant features or strategies, which are then further refined and evaluated by subsequent models. This sequential combination of different outputs ensures a comprehensive and nuanced analysis, leading to more accurate and tailored recommendations for persuasive strategies.

To illustrate this better, we have included a more detailed explanation on the manuscript:

Hence, the fusion strategy in our model, combining different outputs sequentially, was chosen for its efficacy in integrating varied results: the optimal ranking and appropriateness scores for persuasion principles. The first method's ranking filters top strategies, which the second method then refines with scores. This cascade approach, akin to dimensionality reduction simplifying the model's complexity while maintaining

[1] Gómez-Carmona, O., Casado-Mansilla, D., López-de-Ipiña, D., & García-Zubia, J. (2021). Optimizing computational resources for edge intelligence through model cascade strategies. *IEEE Internet of Things Journal*, *9*(10), 7404-7417.

robust predictive power[1], aligns with previous methodologies and is well-suited for handling categorical variables directly. It leverages each model's strengths, sequentially refining the analysis for more precise, user-tailored recommendations.

11. Experiments: The assumption of several analyses is that only one top-ranked strategy per user. In reality, also considering noise, there might be two or more equally suited strategies per use. The evaluation approach thus seems a bit narrow. Could be discussed..

We appreciate the suggestion to consider multiple equally suited strategies per user. In our current approach, we select one top-ranked strategy to focus on the most likely effective strategy for each individual. Why? Because in real-life situations the decision-making of an action is usually binary: we do or we don't. Our way of thinking for an effective strategy is that we must try to enact a behaviour with the strategy that may work best for any single user. This is our rationale and the reason for not addressing the top 3 or top 5. We have included this information in the conclusions of the paper.

However, we acknowledge that this approach may not fully capture the nuances of individual preferences and behaviours (overall for our changing preferences). We expect that our contribution will inspire others to explore the inclusion of multiple suitable strategies for each user, which could provide an assessment of how different strategies might be equally effective in varying contexts.

12. Experimental scenarios: Two datasets are used, leading to different results. A deeper investigation is missing. Also, I wonder if it is meaningful to compare the used metrics on an absolute scale.

Thank you for the feedback. This is again a good point. In the paper, we have now explained better that we tested two approaches to assess the system performance:

- *a) training and testing on the total of both datasets (cross-validation of GreenSoul and Prolific together) N=743,*
- *b) training only with GreenSoul data (N=368) and testing only on the Prolific ones (N=375).*

However, after testing both options, the former outperformed the latter. We explained that the performance drop in the second approach might be because the two datasets represent two different population distributions (even though we did the same type of screening on the Prolific platform). Our conclusion of this comparative experiment was the need to regularly retrain the models with more data to cope with differences in the user characteristics - overall if they wanted to be transferred to different contexts or latitudes. Or even if the people change their mindsets in the short-mid term. What is good about our final user features set is that out of the nine questions, only three might change throughout the time: "Intentions, Confidence in technology, and PST". That was emphasised as well in the discussion section (subsection: Privacy and Ethical considerations and model generalization)

Regarding how we compared the user metrics, we have to say that these absolute values were equally measured in the GreenSoul and the Prolific questionnaire.

13. Figure 3 and the corresponding analysis: Why is the NDPM used here as the only measure? Why are different measures used in the other experiment? Why not reporting F1 here as well?.

Thank you for your insightful query concerning our choice of the Normalized Distance-based Performance Measure (NDPM) in Figure 3 and its corresponding analysis. We appreciate the opportunity to clarify the rationale behind this methodological decision.

The NDPM was chosen because our research primarily focuses on the ordering of answers, a critical aspect in evaluating the effectiveness of our recommendation system. NDPM is adept at measuring the distance between predicted and actual orderings, making it particularly relevant for our study, where the precise ranking of answers reflects the system's performance in predicting user preferences sequentially.

Another key feature of NDPM is its normalisation, which allows for a standardised interpretation of performance across different prediction sets. This feature is essential for our analysis as it provides a more intuitive understanding of how well the system replicates the actual preference order of users.

While metrics like the F1 score are adequate for evaluating classification accuracy, they do not inherently capture the quality of order, which is a crucial component of our system's performance evaluation. Hence, in other experiments focusing on classification accuracy or the presence/absence of items, metrics like F1 score were more applicable. However, for the specific purpose of evaluating the sequential recommendation system in Figure 3, NDPM was the most aligned with our objectives.

14. Conducting a T-SNE to the user embeddings obtained from the model" -> Where do the user embeddings come from here?.

Thank you for your insightful comment and query regarding the origin of user embeddings in our study. We appreciate the opportunity to clarify this aspect of our research.

The user embeddings mentioned in our study are generated by the model employed in it which is implemented using the LightFM library, a hybrid machine learning model that combines traditional matrix factorization (MF) methods with user and item features. In our specific application, the model extends the conventional matrix factorization approach by integrating latent factors related to users and items as is explained in “Proposed method” section.

15. "meaning that the predictions are an average of 1-1.5 units away from the true scores" -> Is this true? It is not the MAE, which can be easily interpreted in absolute terms, but here the authors use the RMSE..

We appreciate your attention to detail and clarification on the interpretation of our results. You are absolutely correct. In the paragraph, we mentioned that "the predictions are an average of 1-1.5 units away from the true scores," which may not accurately represent the RMSE. RMSE indeed measures the square root of the average of the squared errors, so it is not directly interpretable in absolute terms.

We apologize for any confusion caused by our previous statement. To provide a more precise interpretation, we should emphasize that the RMSE values we reported indicate the average magnitude of the prediction errors in relation to the true scores, but they are not straightforwardly comparable to the absolute values of the predictions. Thank you for pointing this out, and we will make sure to clarify this in our manuscript to ensure accuracy and transparency in our reporting.

We revised the document as follows:

> The results of the evaluation are presented in the Figure of RMSE. The horizontal axis contains the target principles, while the vertical axis reports the RMSE errors of predicting the scores for each principle. The lines correspond to the mean RMSE across all cross-validation folds, for the RF and MVR methods, while the error bars correspond to the 95% inter-quantile range. We can see that the RMSE errors range from 1 to 1.5, indicating that the predictions deviate, on average, by 1-1.5 units from the true scores, which fall within the range of 1 to 5. We can also see that the RMSE values vary for all principles, with "Similarity" and "Social proof" achieving the lowest errors, suggesting more accurate predictions for these principles. The errors of the RF method are slightly lower than those of the MVR method, indicating that the RF method is more suitable for predicting individual principles, as expected given that MVR considers all principles simultaneously. However, it's important to note that even though MVR used a rank 5 coefficient matrix, it still achieved relatively high accuracy, especially when focusing on fewer "core" targets.

16. Table 4: Now here we use different forms of the F-score. It is quite surprising that the combination of two rather weak predictors (eg 0.02 and 0.03) leads to a combined value that is multiple times higher (0.11). Unfortunately, no code and data is provided to verify this rather surprising result..

Thank you for your observation regarding the F-score results. The significant improvement in the combined F-score, despite the individual predictors showing weak performance, can indeed seem surprising. Nevertheless, in a cascade of models, it is possible to observe a significant improvement in the combined performance, even when individual models show relatively weak predictive power. This phenomenon occurs because each model in the cascade may capture different aspects or patterns in the data, which, when combined, provide a more comprehensive understanding, enhancing the overall predictive capability of the system. This reflects the strength of ensemble approaches in effectively aggregating diverse predictions to achieve better accuracy.

17. Minor:

- **The writing is a bit wordy at times, starting with a quite long abstract.**
- **Jesse et al. -> Jesse and Jannach**
- **"is low enough to make the model useful in a real setting." -> How do we know?**
- **Starke et al. worked on nudging in the energy efficiency domain, e.g. [a]. Their works could be discussed.**

[a] Alain D. Starke, Martijn C. Willemsen, Chris Snijders: With a little help from my peers: depicting social norms in a recommender interface to promote energy conservation. IUI 2020: 568-578

Thank you for identifying these valuable minor changes suggested below. We have accordingly solved all the addressed issues. We will be responding one by one:

- We have reviewed the manuscript and rewritten part of the abstract to make it less wordy.
- Thank you for pointing out this. We have corrected it.
- This level of accuracy indicates that the model has a reasonable predictive capability, although it is not highly precise. In practical terms, this RMSE range suggests the model can provide useful, albeit not perfect, insights or predictions within the given context. We have emphasized this in the manuscript:

  > In practical terms, this RMSE range suggests the model can provide useful, albeit not perfect, insights or predictions within the given context.

- We want to acknowledge the reviewer for the provided reference. Given its relevance, we have included it in the manuscript in the Discussion section:

  > In this regard, other research works underscore the effectiveness of personalized normative messages in recommender systems for promoting energy conservation [a]. This aligns with the emphasis on contextualized persuasion strategies, substantiating our approach with empirical evidence. Moreover highlights that tailored messages based on social norms can significantly influence user behavior in energy efficiency.

Reviewer 3

1. Discuss what are the implications on different stakeholders- e.g. facility managers (optimized operations, lesser complaints?), occupants (requires more engagement or understanding of system/people), owners (operating costs), etc..

Thank you very much for providing such accurate feedback. We have reworked the discussion section, overall on the implications of the real-time deployment of the system and on the Privacy

and Ethical considerations. For example, regarding policymakers, we explained that our approach was interesting for them as it provided confidence when deciding how to design an intervention based on the reliability of a system or a more collective approach. Besides, it was explained that our approach for the top strategy is very relevant for end-users. Why? Because in real-life situations the decision-making of an action is usually binary: either we do or we don’t. Our way of thinking for an effective strategy is that we must try to enact a behaviour with the strategy that may work best for any single user. This was our rationale and the reason for not testing the top 3 or top 5 in the same person. To conclude, we also explained that the model presented is very important for intervention designers and other scholars. Some individuals would prefer a message such as “your peers are already lowering their energy footprint” (i.e., social proof- People), while others would prefer a message such as “the system allows you to check your energy performance at any time” (i.e., self-monitoring - System). Therefore, we helped to find those nuances and differences. In essence, if instead of looking for the best persuasion strategy, a recommender system would like to know what kind of context is the most valuable to present a message to end-users, the model we presented in the paper can help to provide that with higher reliability which will offset the operating cost of putting in place such a system (without taking into account the energy awareness from occupants).

2. Were there any methods applied to test reporting biases?.

Thank you for your insightful comment. This was also a concern that we should have explained better in the limitation section and on the description of the datasets.
In the paper, we have now explained better that we tested two approaches to assess the system performance:

- *a) training and testing on the total of both datasets (cross-validation of GreenSoul and Prolific together) N=743,*
- *b) training only with GreenSoul data (N=368) and testing only on the Prolific ones (N=375).*

However, after testing both options, the former outperformed the latter. We explained that the performance drop in the second approach might be because the two datasets represent two different population distributions (even though we did the same type of screening on the Prolific platform). But, as suggested by the reviewer there is a learning bias. Our conclusion of this comparative experiment was the need to regularly retrain the models with more data to cope with differences in the user characteristics - overall if they wanted to be transferred to different contexts or latitudes. Or even if the people change their mindsets in the short-mid term. What is good about our final user features set is that out of the nine questions, only three might change throughout the time: “Intentions, Confidence in technology, and PST”. That was also emphasised as well in the discussion section (subsection: Privacy and Ethical considerations and model generalization)

3. How do you envisage this framework can be replicated in specialized settings - such as schools, senior living homes etc..

This is an interesting point. When we designed the framework it was mainly oriented towards office-based workplaces and for building occupants (mainly office employees). In the paper we

discussed that “from a practical point of view, the fact that the proposed method can predict optimal personalised persuasion principles from a few input features makes it suitable for use in real-world applications. In essence, by just obtaining nine features of the users our approach is able to provide bespoke messages to motivate them to continue doing green practices related to energy efficiency in tertiary buildings (e.g. switching off a lamp when not in use, using the low energy mode in their PCs, or nudge people to change equipment to more energy efficiency options). [...] .. in terms of generalization, our model indeed leans on data for a specific field of application (i.e., energy efficiency) and a specific context (i.e., the workplace). Nevertheless, we argue that the model is valid for any intervention related to sustainability issues (e.g., saving water or other resources, or improving the air quality). The only user feature that has to be removed as a predictor is “Work-culture” which was in the fourth position of the most relevant nine features.

4. Can you comment on the micro f-score being slightly better for MVR than RF (for fusion) in Tables 4 and 5?.

Thank you for pointing out the performance differences between MVR and RF as observed in Tables 4 and 5. The slightly superior micro F-score for MVR in the fusion approach could be due to its more effective handling of subtle patterns in the data, and the sensitivity of the micro-F1 metric to the distribution of classes and tend to aggregate the performance across all classes.

In any case, the purpose of this comparison was to highlight the noteworthy small difference in F-scores between MVR and RF (including its micro average). As can be seen, both models achieve comparable overall performance. This outcome highlights the effectiveness of our fusion approach in mitigating the limitations of individual models, even those with lower accuracy despite the factors that may affect the different averaging metrics.

5. Figure 3 What does the x and y axis represent?

Thank you for identifying this aspect. Please, bear in mind that in Figure 3, unlike some other dimensionality reduction techniques (like PCA, where axes can represent principal components), the axes in a t-SNE plot do not have an inherent, interpretable meaning. Thus, they don't correspond to specific original features of the data. In essence, the focus of Figure 3 should be on the relative positioning and clustering of points, rather than trying to interpret the axes themselves. The axes serve as a means to visualize the high-dimensional relationships in a more comprehensible two-dimensional space.
