# Supplementary material for: A cascading model for nudging employees towards energy-efficient behaviour in tertiary buildings
Source: PLoS One. 2024 May 16;19(5):e0303214. doi: 10.1371/journal.pone.0303214 (PMC11098420; doi:10.1371/journal.pone.0303214)
Supplement: Attachment — Submitted filename: 2nd Rebuttal paper PLOS-ONE.pdf [file pone.0303214.s003.pdf]

Responses to reviewers

First of all, we would like to express again our gratitude to the editors and the external reviewers for their valuable and constructive comments on this second round. As in the first one, we found most helpful in improving the overall quality of our paper toward the final version.

We acknowledge the opportunity to submit a new version of this manuscript and now we think it can be considered as final. Thus, we have carefully examined the comments from all reviewers and followed their suggestions. We believe we have addressed all their points in light of their suggestions and comments, which are summarised in the authors' responses below.

To facilitate the reviewing task, we have made all the changes to the manuscript in different colours to differentiate the edited part; **blue** for new additions and **purple** for edited or transformed parts. We refer to them as well in our responses.

Reviewer #2

1. The links to the code repository should be included in the paper.
Thank you for your insightful comments and for recognising the positive outcomes of our research. We understand the importance of transparency and reproducibility in research and agree that providing direct access to the code would greatly facilitate these objectives.

In light of this feedback, we have added the relevant links to the code repository within the paper. The link has been placed in the section where the methods are described, ensuring that readers can easily find and access the code used for our experiments.

https://github.com/morelab/st_recommender

We hope that this amendment addresses the reviewer's concerns and further strengthens the contribution of our paper.

2. There are issues in the GitHub repository: The "models" folder, for example, does not contain models, but some data-related things. I also miss a proper README that describes which code should be run to obtain the results reported in the paper. Given the huge increase in accuracy when combining two models, it might be helpful when a third party can validate this.
Thank you for your feedback. We have considered your comments and have made significant updates to our GitHub repository. We have cleaned the repository in general to avoid misconceptions about the namings. Additionally, we have added a comprehensive README file

that provides detailed instructions on how to run the experiments and replicate the results reported in our paper. We understand the importance of third-party validation, especially given our reported increase in accuracy when combining two models. We encourage you to revisit our repository and review the changes we've made. Your insights are invaluable to us, and we hope that these updates will facilitate a smoother validation process for anyone interested.

3. The new discussion of the relationship between persuasion and nudging needs some backing references. Also, I am not convinced by the statement "Both persuasion and digital nudging involve personalisation". Nudging does not necessarily involve personalization, and persuasion does not either.

Thank you for your insightful comments and reflections on those two interwoven topics. We have rephrased the section and included some important papers that discussed the differences, similarities and nuances of persuasion and nudging.

4. This sentence seems broken: "Over the years, and as occupant behaviour has been vastly recognised as a crucial factor for Energy Efficiency"

We thank the reviewer for raising this error. Accordingly, we have corrected this sentence in the manuscript:

At the same time, over the years, occupant behaviour has been vastly recognised as a crucial factor for Energy Efficiency (EE)

5. Sentence should be revised: "being this consumption behavior particularly"
We acknowledge the reviewer's suggestion for revision. The sentence has ben rephrased for clarity as follows:

Furthermore, it has been identified that buildings occupied by users with wasteful energy behaviour can have twice the consumption as the ones that energy savers generally occupy. *This pattern of consumption behavior is especially pronounced in developing and transition economies.*

6. "behavioral" (page 2, check use of British vs American English)

We have reviewed the manuscript in accordance with your comment and ensured consistency in the use of American English, specifically addressing the use of "behavioral" and “behavior”. Thank you for bringing this to our attention.

7. "Moreover, understanding waste management behavior". What is "waste management behavior"?

Thank you for addressing this lack of clarity. "Waste management behavior" refers to the actions and practices individuals adopt related to the handling of waste, which encompasses reduction,

reuse, recycling, and proper disposal of waste. These behaviors are influenced by a combination of rational calculations of benefits and costs, social pressures, personal beliefs, and attitudes towards environmental conservation.

We have reflected this better in the manuscript:

Moreover, comprehending the nuances of waste management behaviour —which includes the reduction, reuse, recycling, and proper disposal of waste as influenced by rational benefit-cost analyses, social pressures, and personal environmental beliefs and attitudes— plays a pivotal role in enhancing energy efficiency

8. The S1 file is mentioned, but no reference is provided where we can access it. (I could access it through the editorial system, though)
Thank you for flagging this issue of access to content. The S1 is actually in the Zenodo repository which has been several times commented in the manuscript. Nevertheless, I will ask PLOS One assistant and editors about how can make this available. Many thanks in advance.

9. "the Prolific platform 1." -> extra space before footnote
We have corrected this.

10. "they don’t correspond", "However, it’s important to", "or we don’t do" -> contractions like "don't" should be avoided

We have carefully revised the text to eliminate all contractions, ensuring a more formal tone consistent with academic writing standards. Your attention to detail is greatly appreciated.

11. "they don’t correspond", "However, it’s important to", "or we don’t do" -> contractions like "don't" should be avoided
Thank you

Reviewer #3

The authors have addressed all the comments well, the paper can now be accepted in its current form for publication.
